# Two New Cytotoxic Indole Alkaloids from a Deep-Sea Sediment Derived Metagenomic Clone

**DOI:** 10.3390/md12042156

**Published:** 2014-04-08

**Authors:** Xia Yan, Xi-Xiang Tang, Lin Chen, Zhi-Wei Yi, Mei-Juan Fang, Zhen Wu, Ying-Kun Qiu

**Affiliations:** 1 Key Laboratory for Chemical Biology of Fujian Province, School of Pharmaceutical Sciences, Xiamen University, South Xiang-An Road, Xiamen 361102, China; E-Mails: 420912769@qq.com (X.Y.); chenlin13405@126.com (L.C.); fangmj@xmu.edu.cn (M.-J.F.); 2 Key Laboratory of Marine Biogenetic Resources, Third Institute of Oceanography State Oceanic Administration, Xiamen 361005, China; E-Mails: tangxixiang@hotmail.com (X.-X.T.); xyxyyzw@163.com (Z.-W.Y.)

**Keywords:** deep-sea sediment, metagenomic clone, indole alkaloids, metagenetriindole A, metagenebiindole A, cytotoxicity

## Abstract

Two new indole alkaloids, metagenetriindole A (**1**) and metagenebiindole A (**2**), were identified from deep-sea sediment metagenomic clone derived *Escherichia coli* fermentation broth. The structures of new compounds were elucidated by spectroscopic methods. The two new indole alkaloids demonstrated moderately cytotoxic activity against CNE2, Bel7402 and HT1080 cancer cell lines *in vitro*.

## 1. Introduction

Deep marine subsurface sediments are one of the most extensive microbial habitats on Earth. The deep sea is usually characterized by extremely high salinity, darkness, high pressure, and high/low temperature [1]. Due to the particularity of the marine environment, marine microorganisms have unique metabolic properties, producing many novel chemical structures with great complexity and diversity. This untapped potential has resulted in the recent acceleration in interest in the study of marine microorganisms. However, the dilemma is that the vast majority of the microorganisms cannot be cultivated at present [2].

Metagenomics, which utilizes culture-independent methods to access the collective genomes of natural bacterial populations, provides a means of exploring the secondary metabolites produced by the large collections of bacteria that are known to be present in the environment but remain recalcitrant to culturing. The foundation of all metagenomic approaches is the isolation and subsequent examination of DNA extracted directly from naturally occurring microbial populations (environmental DNA, eDNA). The eDNA libraries can be examined in simple high-throughput assays designed to identify clones that associated with the production of bioactive small molecules [3,4].

We previously reported isolation of a new compound, which showed potent analgesic activity on fatty-acid amide hydrolase (FAAH) and monoacylglycerol lipase (MGL), from a deep-sea sediment metagenomic clone [5]. In the continuing studies on deap-sea sediment eDNA libraries, we screen the metagenomic library and found a clone coded QD15 exhibited cytotoxic activity. This paper deals with the isolation, structural elucidation, as well as cytotoxic activities of two new indole alkaloids from the QD15 metagenomic clone derived *E. coli* fermentation broth.

## 2. Results and Discussion

Metagenetriindole A (**1**) was isolated as pink powder and its molecular formula was established as C_26_H_19_N_3_O from a sodiated ion at *m/z* 412 in the ESIMS and further supported by the HRESIMS at 412.1420 (calcd. For C_26_H_19_N_3_ONa, 412.1420) and implying 19 degrees of unsaturation. The presence of amide groups in **1** was evidenced by IR absorption bands at 3270, 1630 cm^−1^. The ^13^C spectrum of **1** showed 26 carbon signals (Table 1), which were assigned by the assistance of DEPT spectrum to 15 sp^2^ methines, one sp^3^ methine and 10 sp^2^ quaternary carbons. The ^1^H NMR spectrum displayed an olefinic proton at δ_H_ 8.74 (1H, d, *J* = 3.2 Hz), a set of four-spin proton system signals at δ_H_ 7.43 (1H, br. d, *J* = 7.3 Hz), 8.19 (1H, br. d, *J* = 7.3 Hz), 7.13 (1H, td, *J* = 7.1, 1.2 Hz) and 7.17 (1H, td, *J* = 7.1, 1.2 Hz), as well as a down field labile proton singlet at δ_H_ 11.95 (1H, br. d, *J* = 2.5 Hz), suggested that **1** might have a 3-substituted indole moiety. Another set of proton signals belonging to two symmetrical *mono*-substituted indole moieties could also be found in ^1^H NMR spectrum at δ_H_ 7.24 (2H, d, *J* = 2.2 Hz), 7.65 (2 H, br. d, *J* = 8.1 Hz), 7.31 (2H, br. d, *J* = 8.1 Hz), 6.90 (2H, td, *J* = 8.1, 1.0 Hz), 7.02 (2H, td, *J* = 7.1, 1.0 Hz) and 10.86 (2H, br. d, *J* = 1.7 Hz). The ^13^C NMR spectrum also revealed the presence of the three indole rings, at δ_C_ 133.8 (C), 126.0 (C), 115.6 (CH), 112.0 (CH), 121.6 (CH), 122.7 (CH), 121.5 (CH), 136.6 (C) and δ_C_ 124.0 (2CH), 114.3 (2C), 126.7 (2C), 119.3 (2CH), 118.2 (2CH), 120.8 (2CH), 111.3 (2CH), 136.1 (2C). Other signals in the ^1^H and ^13^C NMR spectra indicated the presence of a methane in high field at δ_H_ 6.41 (1 H, s) and δ_C_ 42.7 (CH), as well as a carbonyl in low field at δ_C_ 193.9 (C). The attachment of carbonyl to a double bond sp^2^ carbon and an sp^3^ carbon, could be deduced from the chemical shift at δ_C_ 193.9 (C). Hence, the 3-C of an indole ring, together with the unique sp^3^ methine, was linking to the carbonyl.

**Table 1:** ^1^H (400 MHz, DMSO-*d*_6_) and ^13^C (100 MHz, DMSO-*d*_6_) NMR data, ^1^H-^1^H COSY and HMBC correlations for **1**.

Position	δ_H_ (*J* in Hz)	δ_C_, Multiple		^1^H-^1^H COSY	HMBC
1		193.9	C		
2	6.41 s	42.7	CH		C-1, 3′, 3″, 3‴, 4″, 4‴
1′	11.95 br.d (2.5)			H-2′	n.o. ^a^
2′	8.74 d (3.2)	133.8	CH	H-1′	C-3′, 3a′, 7a′
3′		126	C		
3a′		115.6	C		
4′	7.43 br.d (7.3)	112	CH	H-5′	C-3′, 6′, 7a′
5′	7.13 td (7.1, 1.2)	121.6	CH	H-4′, H-6′	C-3a′, 7′
6′	7.17 td (7.1, 1.2)	122.7	CH	H-5′, H-7′	C4′, 7a′
7′	8.19 br.d (7.3)	121.5	CH	H-6′	C-3a′, 6′, 7a′
7a′		136.6	C		
1″, 1‴	10.86 br.d (1.7)			H-2″, 2‴	
2″, 2‴	7.24 br.d (2.2)	124	CH	H-1″, 1‴	C-3″, 3a″, 7a″, C-3‴, 3a‴, 7a‴
3″, 3‴		114.3	C		
3a″, 3a‴		126.7	C		
4″, 4‴	7.65 br.d (8.1)	119.3	CH	H-5″, 5‴	C-3″, 6″, 7a″, C-3‴, 6‴, 7a‴
5″, 5‴	6.90 td (8.1, 1.0)	118.2	CH	H-4″, 4‴, H-6″, 6‴	C-3a″, 7″, C-3a‴, 7‴
6″, 6‴	7.02 td (7.1, 1.0)	120.8	CH	H-5″, 5‴, H-7″, 7‴	C4″, 7a″, C4‴, 7a‴
7″, 7‴	7.31 br.d (8.1)	111.3	CH	H-6″, 6‴	C-3a″, 6″, 7a″, C-3a‴, 6‴, 7a‴
7a″, 7a‴		136.1	C		

^a^ n.o. is not observed.

**Figure 1:**
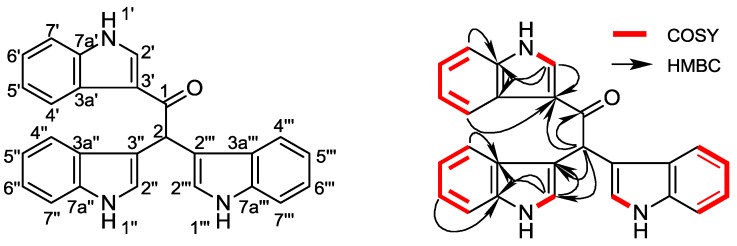
Structure and key ^1^H-^1^H COSY, HMBC correlations of metagenetriindole A (**1**).

From the ^1^H-^1^H COSY spectrum of **1**, the separate spin systems of H-1′/H-2′, H-1″, 1‴/H-2″, 2‴, H-4′/H-5′/H-6′/H-7′, and H-4″, 4‴/H-5″, 5‴/H-6″, 6‴/H-7″, 7‴ were differentiated. These data, together with the HMBC correlations among H-2′/C-3a′, C-7a′ and H-2″, 2‴/C-3a″, 3a‴, C-7a″, 7a‴, confirmed the structure fragments of the three indole rings. However, the molecular framework was finally established by the HMBC correlations between H-2 (δ_H_ 6.41) and C-1, C-3′, C-3″, 3‴, C-4″, 4″, as shown in Figure 1. Up till now, the structure of **1** was elucidated as 1,2,2-tri-1*H*-indol-3-ylethanone, named metagenetriindole A. Similar tri-indole type compounds have also been reported previously, especially from metagenomic clones. Gillespie *et al.* isolated turbomycin A from clones P57G4, *E. coli* with DNA extracted directly from soil, in which the three indole rings are connected to a methyl instead of ethanone [6]. To the best of our knowledge, this is the first report of the isolation of a tri-indole type new compound from metagenomic library derived from deep-sea sediment [7].

Metagenediindole A (**2**) was isolated as a yellow powder and ESI-MS gave its quasi-molecular ion peak at *m/z* 263 [M + H]^+^, 285 [M + Na]^+^. The molecular formula was established as C_17_H_14_N_2_O by the HRESIMS at 285.0995 (calcd. For C_17_H_14_N_2_ONa, 285.0998). The ^1^H and ^13^C NMR spectra indicated that **2** was also an indole type compound. In the ^1^H NMR spectrum, an entire set of 3′-substituted indole proton signals, including a set of four-spin proton system signals at δ_H_ [7.10 (1H, br.d, *J* = 8.1 Hz), 6.80 (1H, td, *J* = 8.1, 1.0 Hz), 7.01 (1H, td, *J* = 7.1, 1.0 Hz), 7.32 (1H, br.d, *J* = 8.1 Hz)], a down field labile proton singlet at δ_H_ 11.03 (1H, br. s), as well as an olefinic proton at δ_H_ 7.37 (1H, d, *J* = 2.4 Hz), could be observed. Another *ortho*-substituted benzene ring proton signal at δ_H_ 7.44 (1H, br.d, *J* = 7.3 Hz), 6.73 (1H, td, *J* = 7.1, 0.5 Hz), 7.50 (1H, td, *J* = 8.3, 1.3 Hz) and 6.90 (1H, br.d, *J* = 8.3 Hz), could also be found, showing the existence of another indole ring. However, the olefinic proton signal at H-2 position of the indole ring was absent, and the amine labile proton singlet was high-field shifted to δ_H_ 7.73 (1H, br. s), suggesting that the C-2, 3 double bond in this indole ring was substituted by other functional groups. The carbon signals of the complete 3′-substituted indole ring and the *ortho*-substituted benzene ring, could be observed in the ^13^C spectrum of **2**. A carbonyl signal at δ_C_ 203.3 (C) and an sp^3^ quaternary carbon signal at δ_C_ 65.0 (C), were attributed to the C-3 and C-2 carbon of the other indole ring. Otherwise, a methyl signal at δ_H_ 1.62 (3H, s) and δ_C_ 65.0 (C) was furnished in the ^1^H and ^13^C spectrum, respectively.

**Figure 2:**
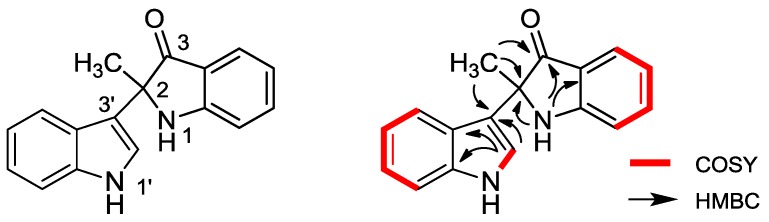
Structure and key ^1^H-^1^H COSY, HMBC correlations of metagenediindole A (**2**).

From the ^1^H-^1^H COSY spectrum of **2**, the separate spin systems of H-1′/H-2′, H-4′/H-5′/H-6′/H-7′, and H-4/H-5/H-6/H-7 were differentiated, which confirmed the structure fragments of the two indole rings. The molecular framework was finally established by the HMBC correlations between methyl proton signal (δ_H_ 1.62) and C-2, C-3, C-3′, as shown in Figure 2. Up till now, the structure of **2** was elucidated as 2-methyl-1,2-dihydro-1′*H*,3*H*-2,3′-biindol-3-one, named metagenediindole A, as a new di-indole type compound from deep-sea sediment metagenomic library. Although the chiral center at C-2 suggested the optical activity of the compound, measured optical rotation value was nearly zero, indicating that compound **2** was isolated as a pair of externally compensated compounds. Similar compound has also been found from metagenome. Abe *et al.* constructed a metagenomic library from the marine sponge *Halichondria okadai* and screened for colored clones then separated a novel compound halichrome A (1), which was different from metagenediindole A (**2**) with C-2 substituted by ethyl [8]. 

The cytotoxic activities of these compounds were preliminarily evaluated using CNE2, Bel7402 and HT1080 cell lines by the CCK 8 method [9]. The results revealed that the two new indole alkaloids, exhibited moderately cytotoxic activities against CNE2, Bel7402 and HT1080 cell lines with IC_50_ values 47.70, 50.55, 44.58 μg/mL (**1**) and 34.25, 43.62, 35.80 μg/mL (**2**) respectively.

## 3. Experimental Section

### 3.1. General Experimental Procedures

UV spectra were recorded on a Shimadzu UV-260 spectrometer (Shimadzu, Tokyo, Japan). IR spectra were determined on a Perkin-Elmer 683 (Perkin-Elmer, Norwalk, CT, USA) infrared spectrometer in KBr pellets. NMR spectra were taken with TMS as internal standard on a Bruker Avance 400 FT-NMR spectrometer (Bruker, Bremen, Germany). ESI-MS were measured on an AB MD-SCIEX Advantage spectrometer (Applied Biosystems, Foster, CA, USA) and HR-ESI-MS on a Bruker FT-MS Apex III spectrometer (Bruker, Bremen, Germany). Column chromatography was performed on silica gel (Yantai Chemical Industry Research Institute, Yantai, China) and Cosmosil 75 C_18_-OPN (75 μm, Nakalai Tesque Co. Ltd., Kyoto, Japan). TLC was conducted on Silica GF254 (Yantai Chemical Industry Research Institute, Yantai, China) and RP-18 F254 (Merck, Scandici, Germany) plates. Detection was done by spraying 10% H_2_SO_4_/ethanol, followed by heating. HPLC was performed with a Varian preparative apparatus using an ODS column (Shimadzu C_18_, 20 × 250 mm, Kyoto, Japan).

### 3.2. Fermentation

Sediment samples for DNA extraction were collected from the subsurface sediments at water depths of 3006 m (102.612575°E, 2.022449°N) in Southwestern Indian Ocean by the Third Institute of Oceanography of China. The samples were maintained at 4 °C before being processed. Sediment samples were precultured in 2216E medium for three days, then, the DNA of enrichment product were extracted and purified, and the size-separated DNAs of 30–40 kb were pooled and end-repaired to blunt end and cloned into fosmid vector. The ligation mixture was packed, and the packaged DNA was transformed into *E. coli*, generating a library of 3500 clones, followed by cytotoxic activity screening. The clone QD15 producing light green pigment was selected and characterized. Finally, QD15 was vaccinated into 100 L fermentation tank and fermented for 36 h at 37 °C, 200 rpm, pH 7.0. Fermentation broth supernatant fluid was collected by continuous flow centrifuge at 60 L/min throughput. A voucher specimen (QD15) has been deposited at the Third Institute of Oceanography, State Oceanic Administration of China.

### 3.3. Extraction and Isolation

AB-8 macroporous adsorption resin (5 L) was used to handle the fermentation broth (80 L). After eluted with 15 L water, the 95% ethanol eluents (15 L) were collected, followed by evaporating the solvent under reduced pressure to give the total extract (108.7 g). The total extract was extracted with ethyl acetate (EtOAc). The EtOAc extract left after removal of the solvent (18.5 g) was separated by silica gel and eluted using a mixture of chloroform/methanol in a stepwise fashion from 100:1—pure methanol to yield 20 fractions. Fraction 10 was chromatographed on ODS column and eluted using a mixture of methanol and water (10:90, 30:70, 50:50, 70:30, 90:10, pure methanol) to yield 18 subfractions. Fraction 10-10 was purified by RP-HPLC, using a mixture of methanol and water in a stepwise manner from 40:60 to 60:40, to yield **1** (2.0 mg) and **2** (1.5 mg).

Metagenetriindole A (**1**): Pink powder; ^13^C NMR (100 MHz, DMSO-*d_6_*) and ^1^H NMR (400 MHz, DMSO-*d_6_*) spectral data were listed in Table 1; ESIMS: *m/z* 389 [M + H]^+^, 412 [M + Na]^+^; HR-ESI-MS: *m*/*z* 412.1420 (calcd. For C_26_H_19_N_3_ONa, 412.1420) (Supplementary Information, Figures S1, S2, S4, S6 and S7).

Metagenediindole A (**2**): Yellow powder; ^13^C NMR (100 MHz, DMSO-*d_6_*) and ^1^H NMR (400 MHz, DMSO-*d_6_*) spectral data were listed in Table 2; ESIMS: *m/z* 263 [M + H]^+^, 285 [M + Na]^+^; HR-ESI-MS: *m*/*z* 285.0995 (calcd. For C_17_H_14_N_2_ONa, 285.0998) (Supplementary Information, Figures S3, S5 and S8–S14).

**Table 2:** ^1^H (400 MHz, DMSO-*d*_6_) and ^13^C (100 MHz, DMSO-*d*_6_) NMR data, ^1^H-^1^H COSY and HMBC correlations for **2**.

Position	δ_H_ (*J* in Hz)	δ_C_, Multiple		^1^H-^1^H COSY	HMBC
1	7.73 br.s				C-2, 3, 3a
2		65.0	C		
3		203.3	C		
3a		117.7	C		
4	7.44 br.d (7.3)	124.3	CH	H-5	C-6, 7a
5	6.73 td (7.1, 0.5)	117.1	CH	H-4, H-6	C-7
6	7.50 td (8.3, 1.3)	137.5	CH	H-5, H-7	C-4, 7a
7	6.90 br.d (8.3)	111.9	CH	H-6	C-5
7a		160.5	C		
1′	11.03 br.s.			H-2′	n.o.
2′	7.37 d (2.4)	123.5	CH	H-1′	C-2′, 3a′, 7a′
3′		114.4	C		
3a′		124.8	C		
4′	7.10 br.d (8.1)	119.5	CH	H-5′	C-7a′
5′	6.80 td (8.1, 1.0)	118.5	CH	H-4′, H-6′	C-3a′, 7′
6′	7.01 td (7.1, 1.0)	121.0	CH	H-5′, H-7′	C-5′, 7a′
7′	7.32 br.d (8.1)	111.6	CH	H-6′	C-3a′
7a′		136.6	C		
2-CH_3_	1.62 s	38.0	CH		C-2, 3, 3′

^a^ n.o. is not observed.

### 3.4. Cytotoxic Activity

The cytotoxic activities of these compounds were preliminarily evaluated using CNE2, Bel7402 and HT1080 cell lines by the CCK8 method [9]. Human nasopharyngeal cells CNE2, hepatoma cells BEL7402 and osteosarcoma cells HT1080 (all purchased from CCTCC, Wuhan, China) grown in DMEM (Dulbecco Modified Eagle Medium) supplemented with 10% fetal bovine serum and 1% (w/v) penicillin/strepto-mycin were seeded as 100 μL aliquots into a sterile 96 well microtiter plate at a titer of approximately 1000 cells per plate and incubated (24 h, 37 °C, 5% CO2). Compounds **1** and **2** resuspended in DMSO and a compound-free DMSO control were diluted in fresh medium and added to the appropriate wells at final concentrations of 100, 50, 25, 13, 6.3, 3.1, 1.6, 0.78, 0.39 and 0.20 μg/mL. These plates were then cultured for an additional 72 h. A cell counting kit 8 (CCK8) assay was used to assess the cytotoxicity of compound 1 and 2 to the cells. Briefly, 10 μL CCK8 solution (Dojindo Laboratories, Kumamoto, Japan) was added to each well and the 96-well plate was continuously incubated at 37 °C for 2 h. The OD value for each well was read at a 450 nm wavelength to determine the cell survival rate on a microplate reader (Epoch; Biotek, Winooski, VT, USA). The assay was repeated three times. An IC50 value was calculated using Origin 7.5 software (OriginLab, Northampton, MA, USA).

## 4. Conclusions

Metagenomics provides a means of exploring the secondary metabolites produced by the large collections of bacteria that are present in such extreme environment as deep sea, which are remain recalcitrant to culturing. Novel bioactive small molecules have been identified using metagenomic approaches in recent studies [10,11]. We screened a 3500 clones including deep sea origin eDNA library and found a cytotoxic activity clone, QD15. Further investigation on the chemical constituents of QD15 clone, lead to the identification of two new indole alkaloids, metagenetriindole A (**1**) and metagenediindole A (**2**), which were found to exhibited moderately cytotoxic activities against CNE2, Bel7402 and HT1080 cell lines. To the best of our knowledge, the two novel compounds is the first reported cytotoxic compound derived from deep sea metagenomic.

## Supplementary Files

Supplementary File 1 Supplementary Information (PDF, 936 KB)
