# Supplementary material for: Two New Cytotoxic Indole Alkaloids from a Deep-Sea Sediment Derived Metagenomic Clone
Source: Mar Drugs. 2014 Apr 8;12(4):2156–63. doi: 10.3390/md12042156 (PMC4012447; doi:10.3390/md12042156)

Supplementary Information

Figure S1. Positive HRESIMS of compound **1**.
Figure S2. ^{1}H NMR spectrum (400 MHz) of compound **1** in DMSO-d_6.
Figure S3. ^{13}C NMR spectrum (100 MHz) of compound **2** in DMSO-d_6.
Figure S4. DEPT spectrum (100 MHz) of compound **1** in DMSO-d_6.
Figure S5. HSQC spectrum (400 MHz) of compound **2** in DMSO-d_6.
Figure S6. ^{1}H-^{1}H COSY spectrum (400 MHz) of compound **1** in DMSO-d_6.
Figure S7. HMBC spectrum (400 MHz) of compound **1** in DMSO-d_6.
Figure S8. Positive ESIMS of compound **2**.
Figure S9. ^{1}H NMR spectrum (400 MHz) of compound **2** in DMSO-d_6.
Figure S10. ^{13}C NMR spectrum (100 MHz) of compound **2** in DMSO-d_6.
Figure S11. DEPT spectrum (100 MHz) of compound **2** in DMSO-d_6.
Figure S12. HSQC spectrum (400 MHz) of compound **2** in DMSO-d_6.
Figure S13. ^{1}H-^{1}H COSY spectrum (400 MHz) of compound **2** in DMSO-d_6.
Figure S14. HMBC spectrum (400 MHz) of compound **2** in DMSO-d_6.

Figure S1. Positive HRESIMS of compound **1**.

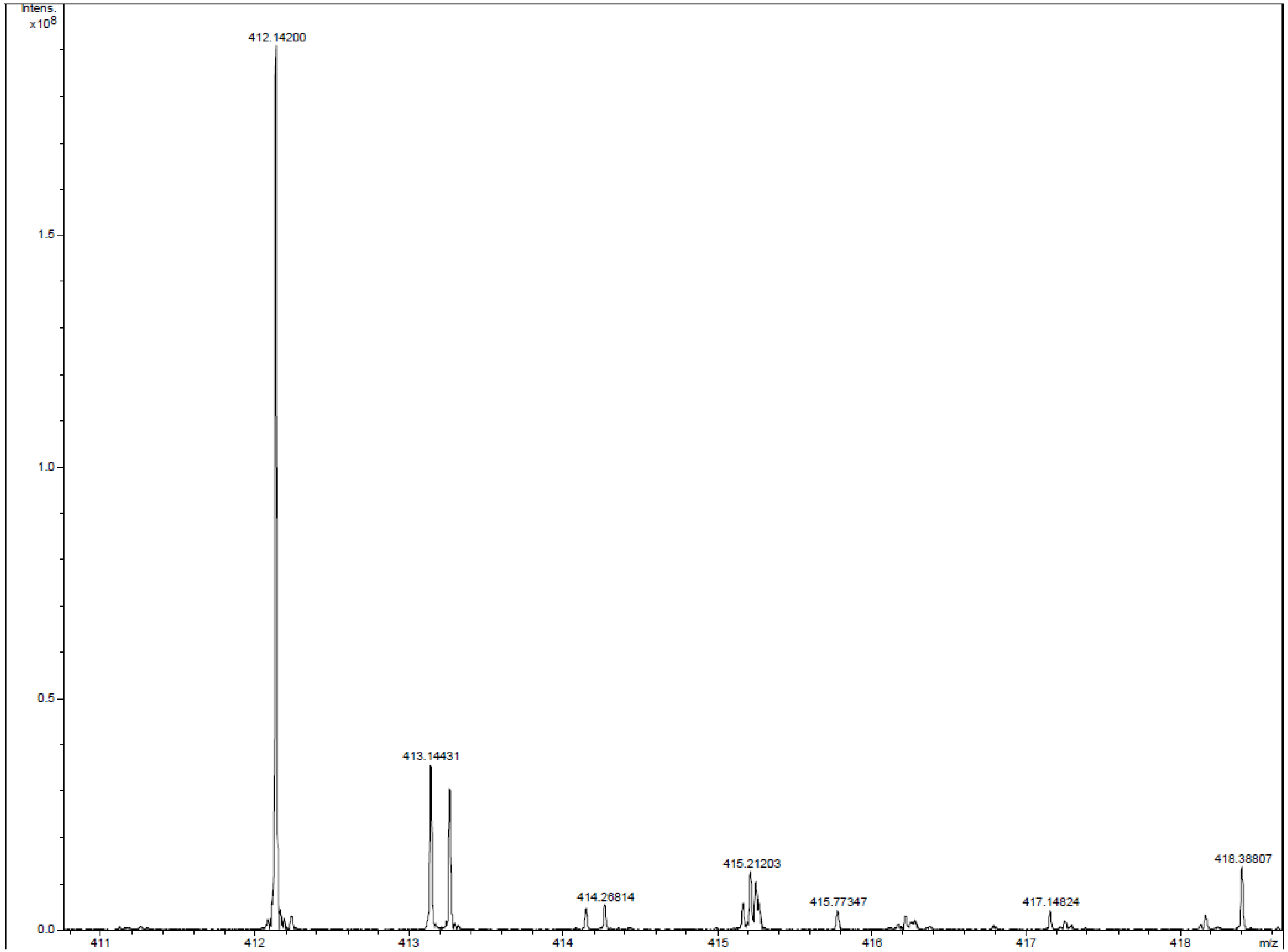

Figure S2. ^{1}H NMR spectrum (400 MHz) of compound **1** in DMSO-d_6.

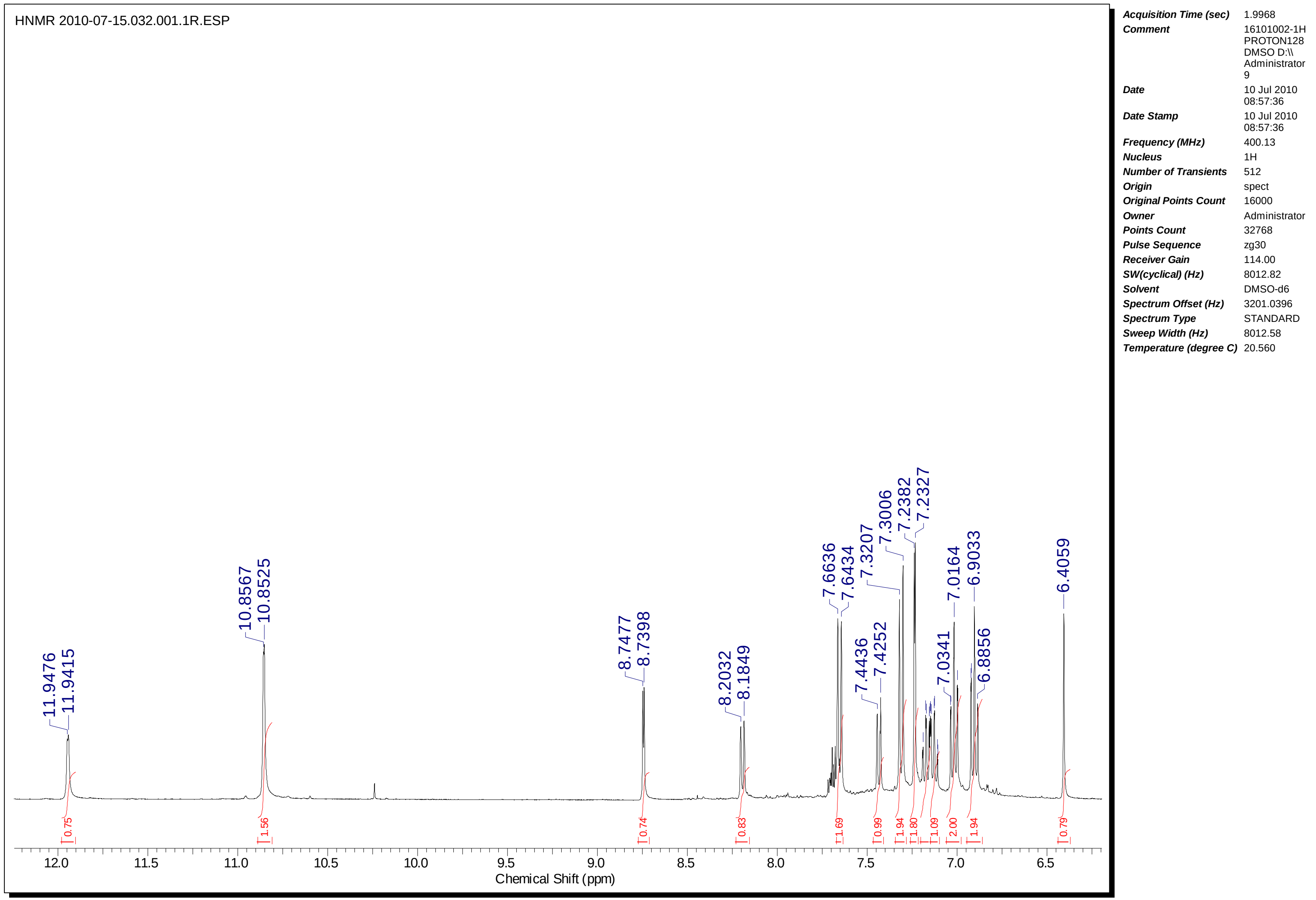

Figure S3. ^{13}C NMR spectrum (100 MHz) of compound **2** in DMSO-d_6.

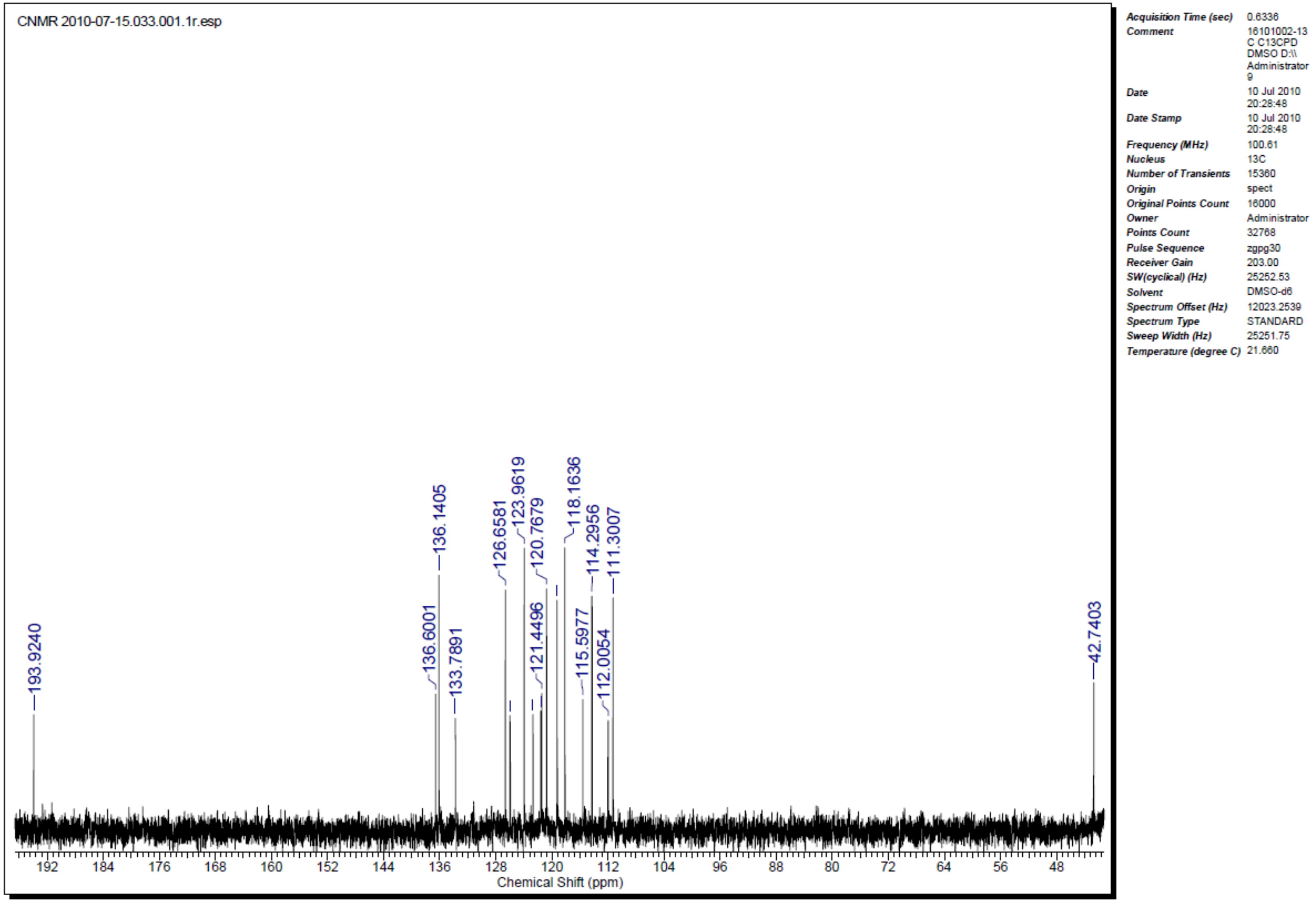

Figure S4. DEPT spectrum (100 MHz) of compound **1** in DMSO-d_6.

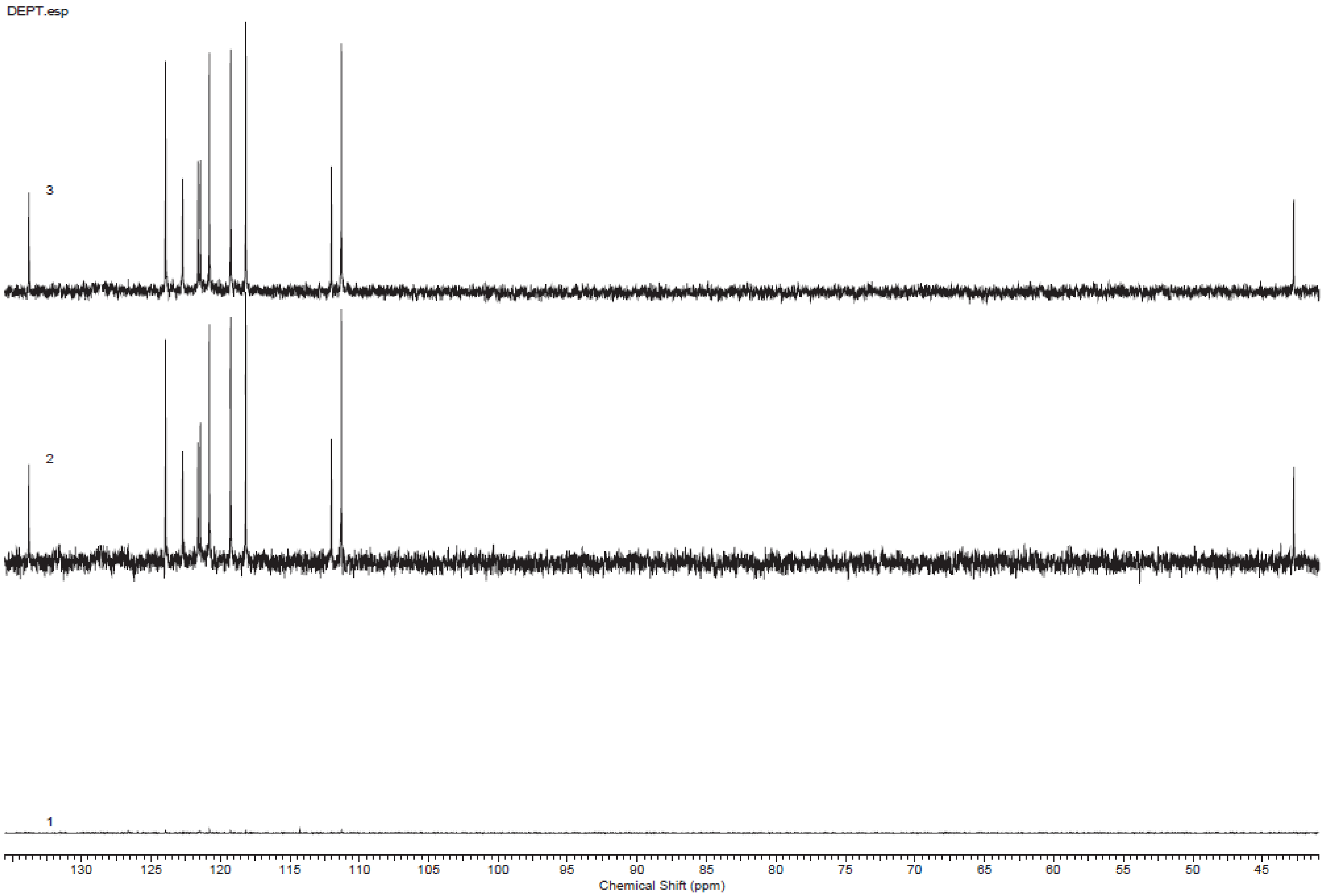

Figure S5. HSQC spectrum (400 MHz) of compound **2** in DMSO-d_6.

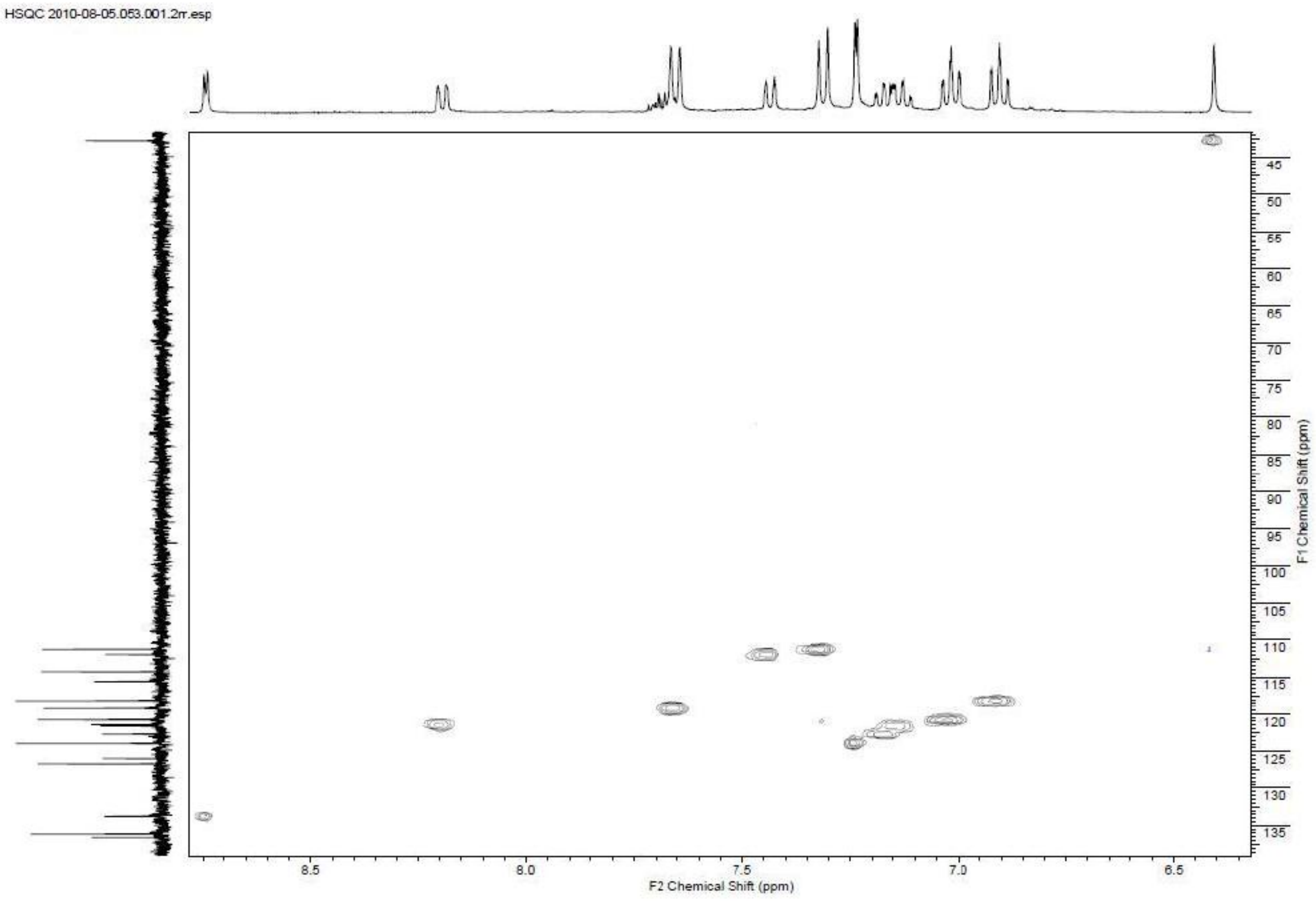

Figure S6. ^1H-^1H COSY spectrum (400 MHz) of compound **1** in DMSO-d_6.

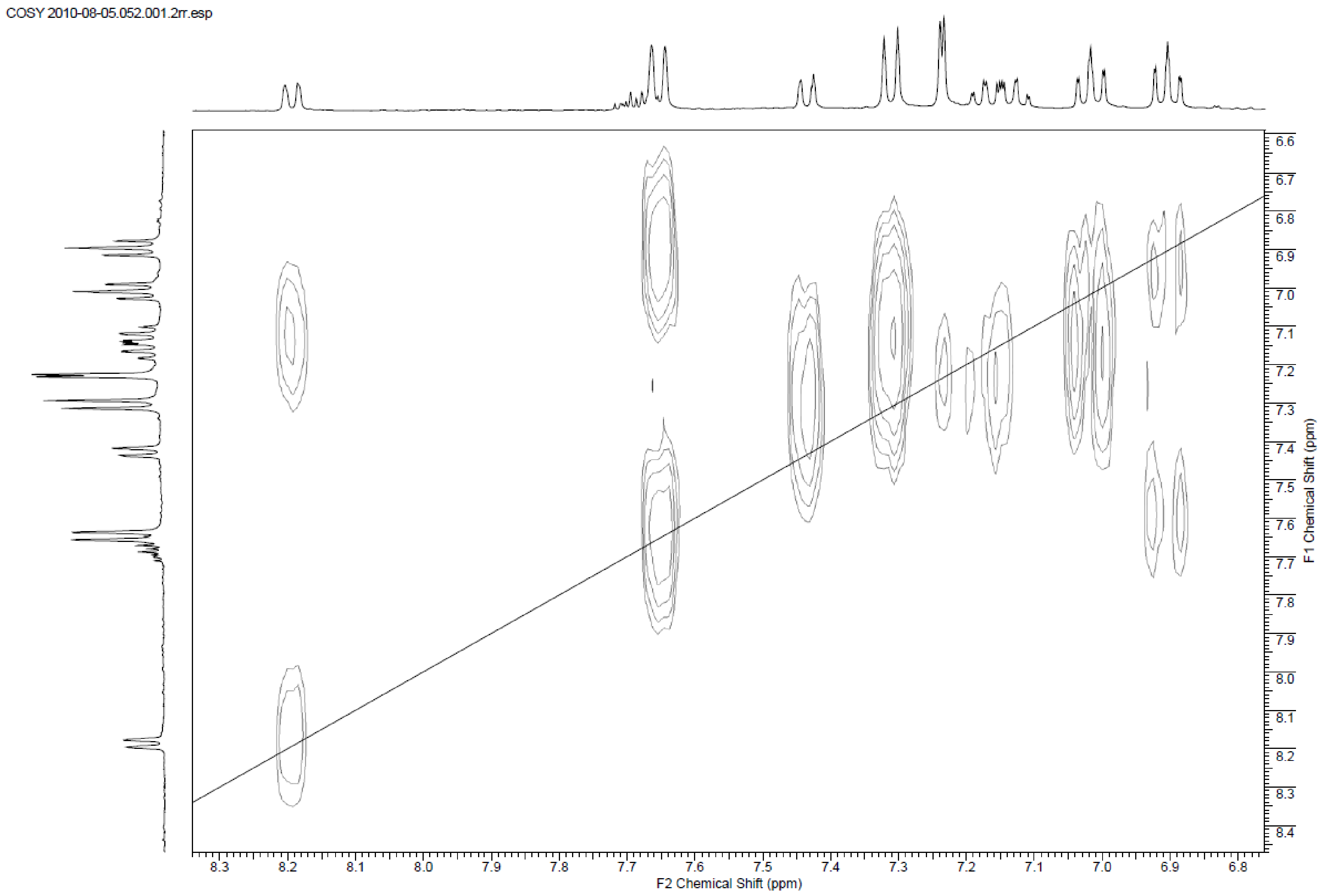

Figure S7. HMBC spectrum (400 MHz) of compound **1** in DMSO-d_6.

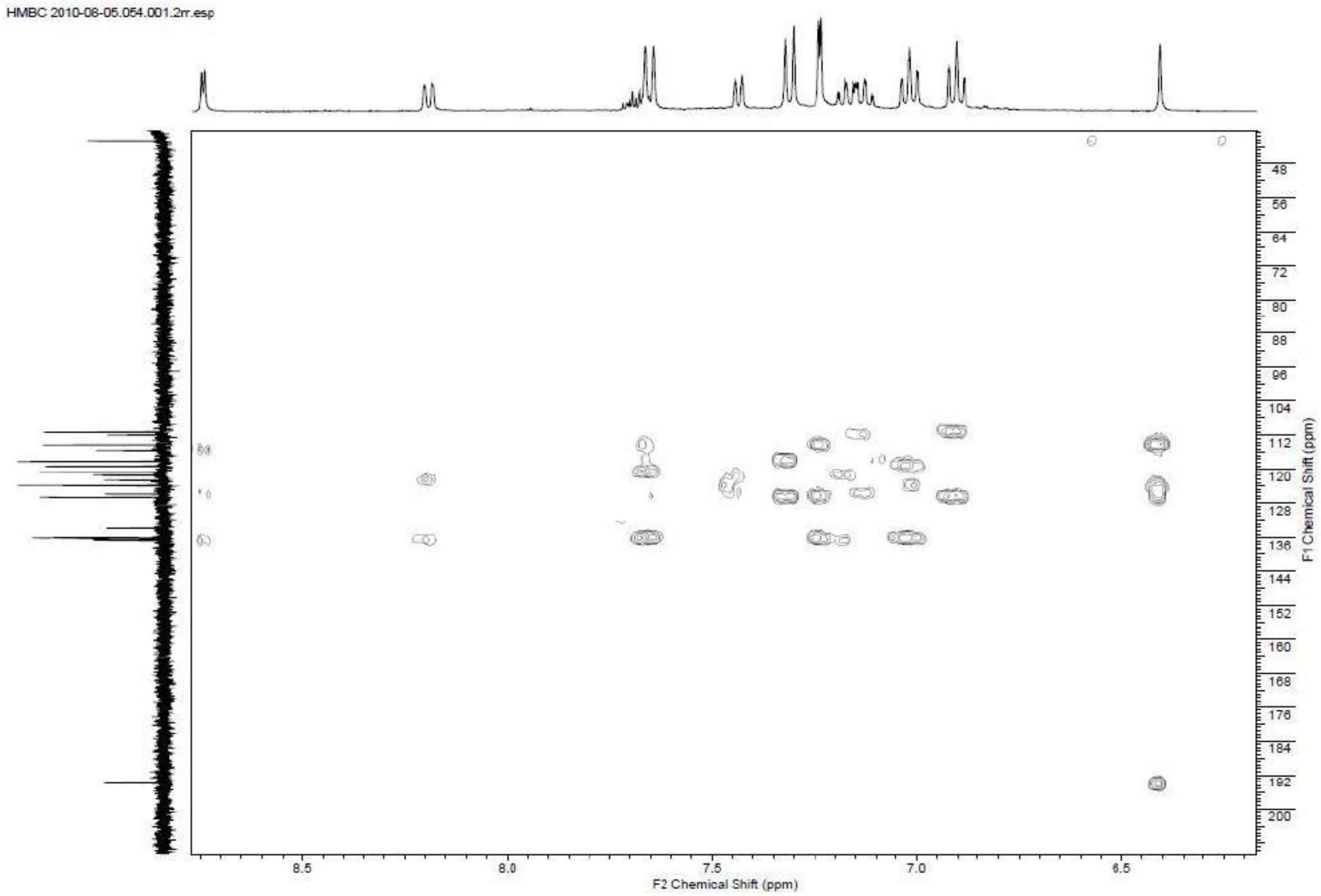

Figure S8. Positive ESIMS of compound **2**.

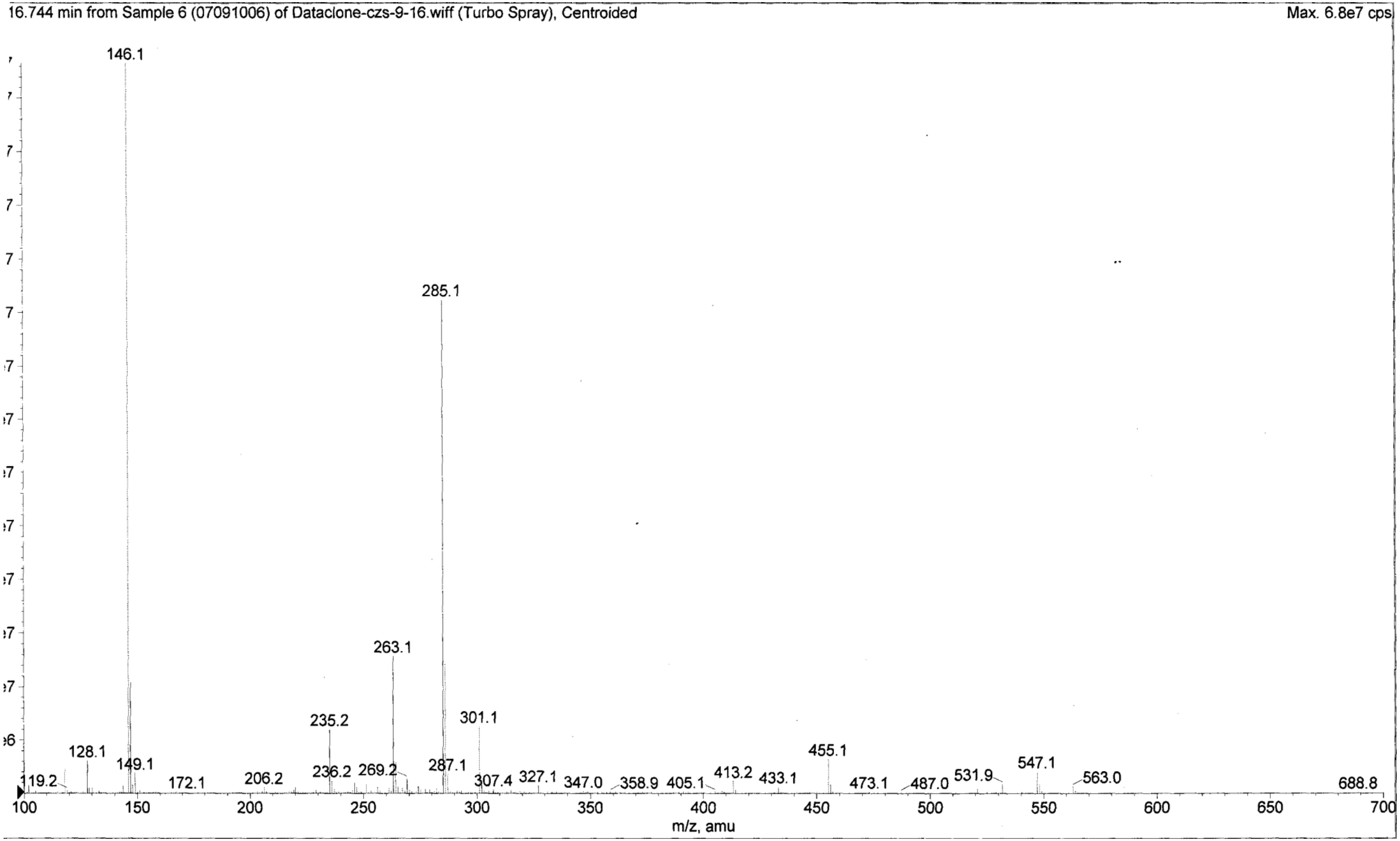

Figure S9. ^{1}H NMR spectrum (400 MHz) of compound **2** in DMSO-d_6.

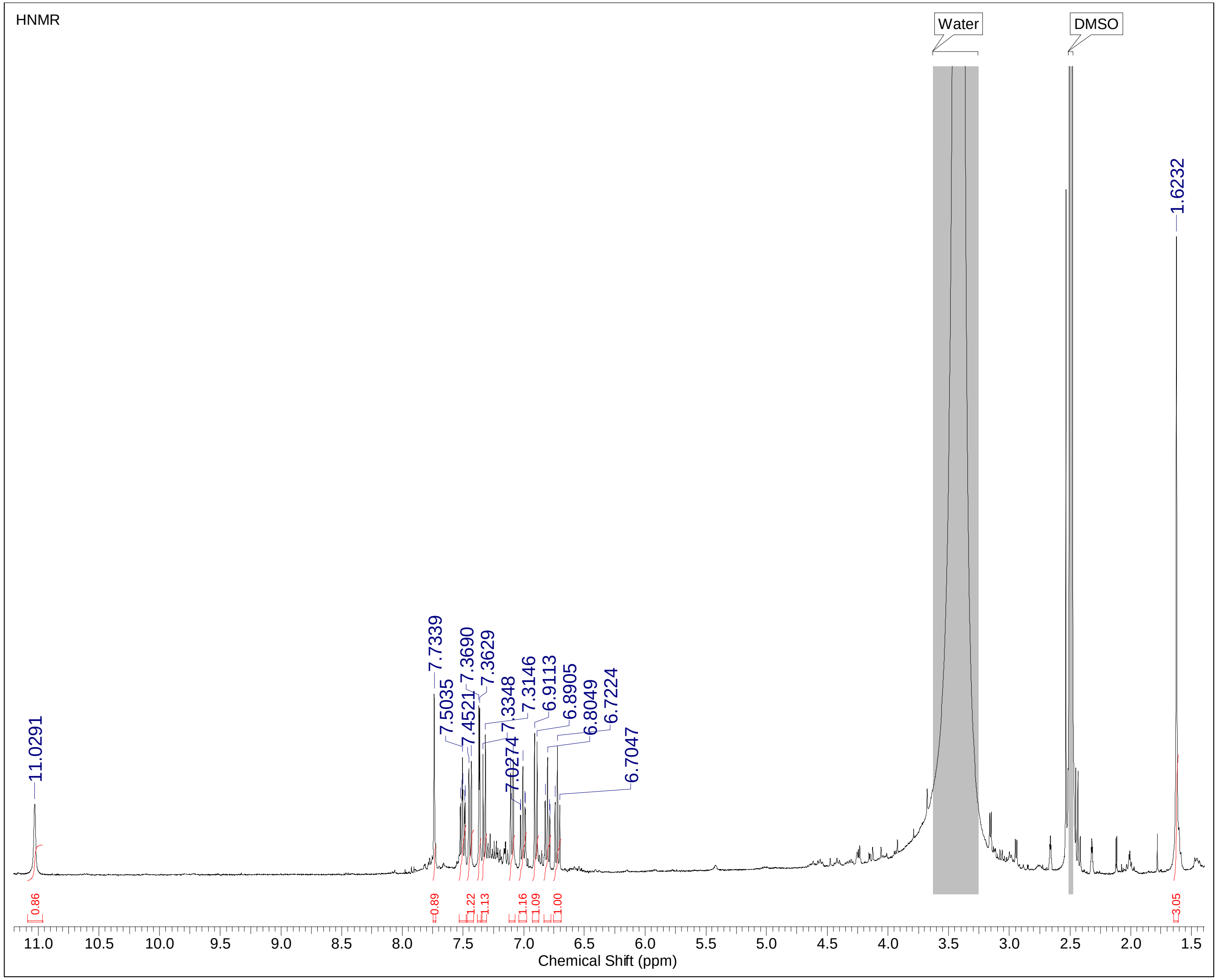

Acquisition Time (sec)	1.9968
Comment	07091006-1H PROTON128 DMSO D:\\ Administrator 3
Date	13 Jul 2010 08:57:36
Date Stamp	13 Jul 2010 08:57:36
Frequency (MHz)	400.13
Nucleus	1H
Number of Transients	256
Origin	spect
Original Points Count	16000
Owner	Administrator
Points Count	32768
Pulse Sequence	zg30
Receiver Gain	114.00
SW(cyclical) (Hz)	8012.82
Solvent	DMSO-d6
Spectrum Offset (Hz)	3201.0396
Spectrum Type	STANDARD
Sweep Width (Hz)	8012.58
Temperature (degree C)	20.960

Figure S10. ^{13}C NMR spectrum (100 MHz) of compound **2** in DMSO-d_6.

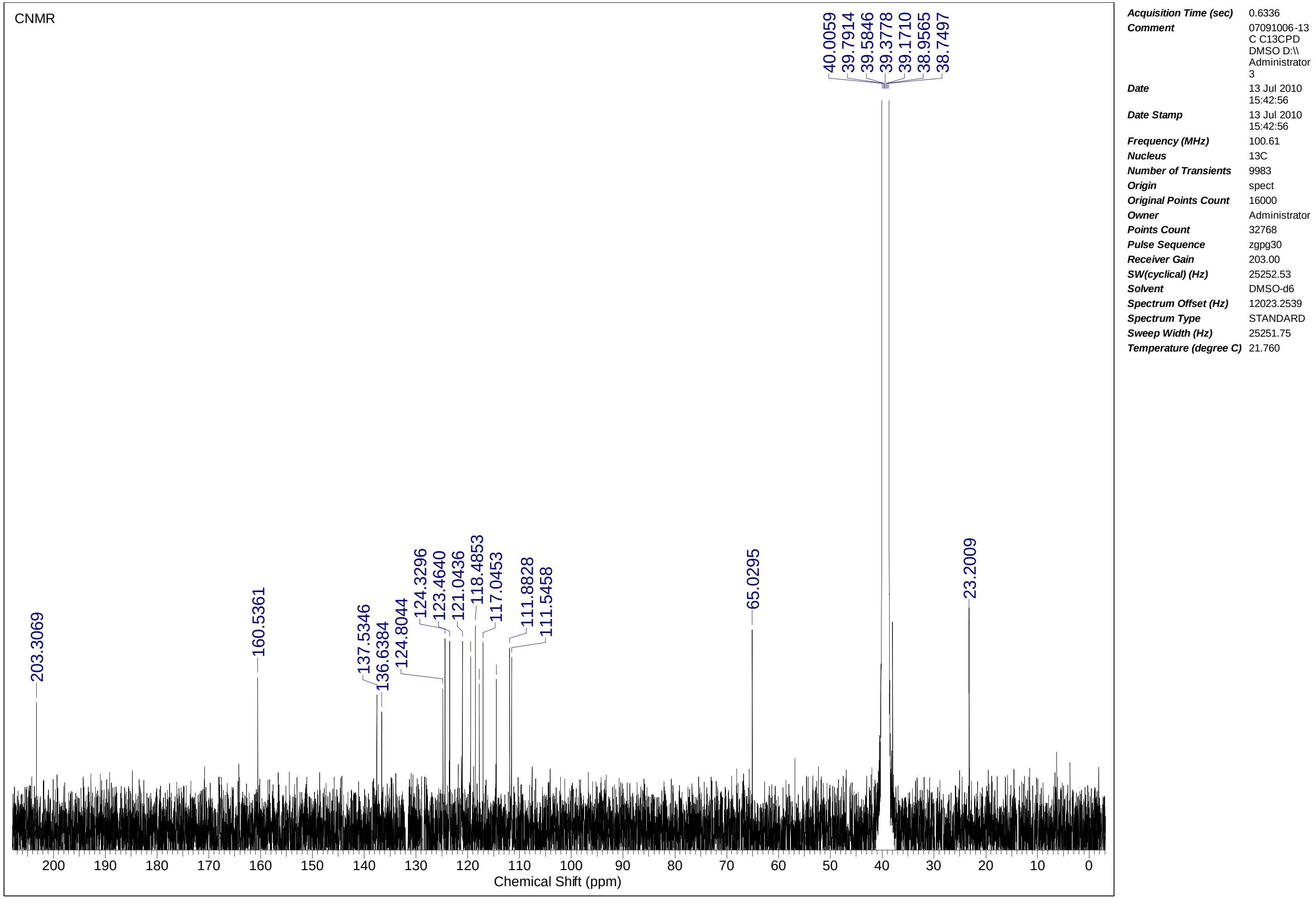

Figure S11. DEPT spectrum (100 MHz) of compound **2** in DMSO-d_6.

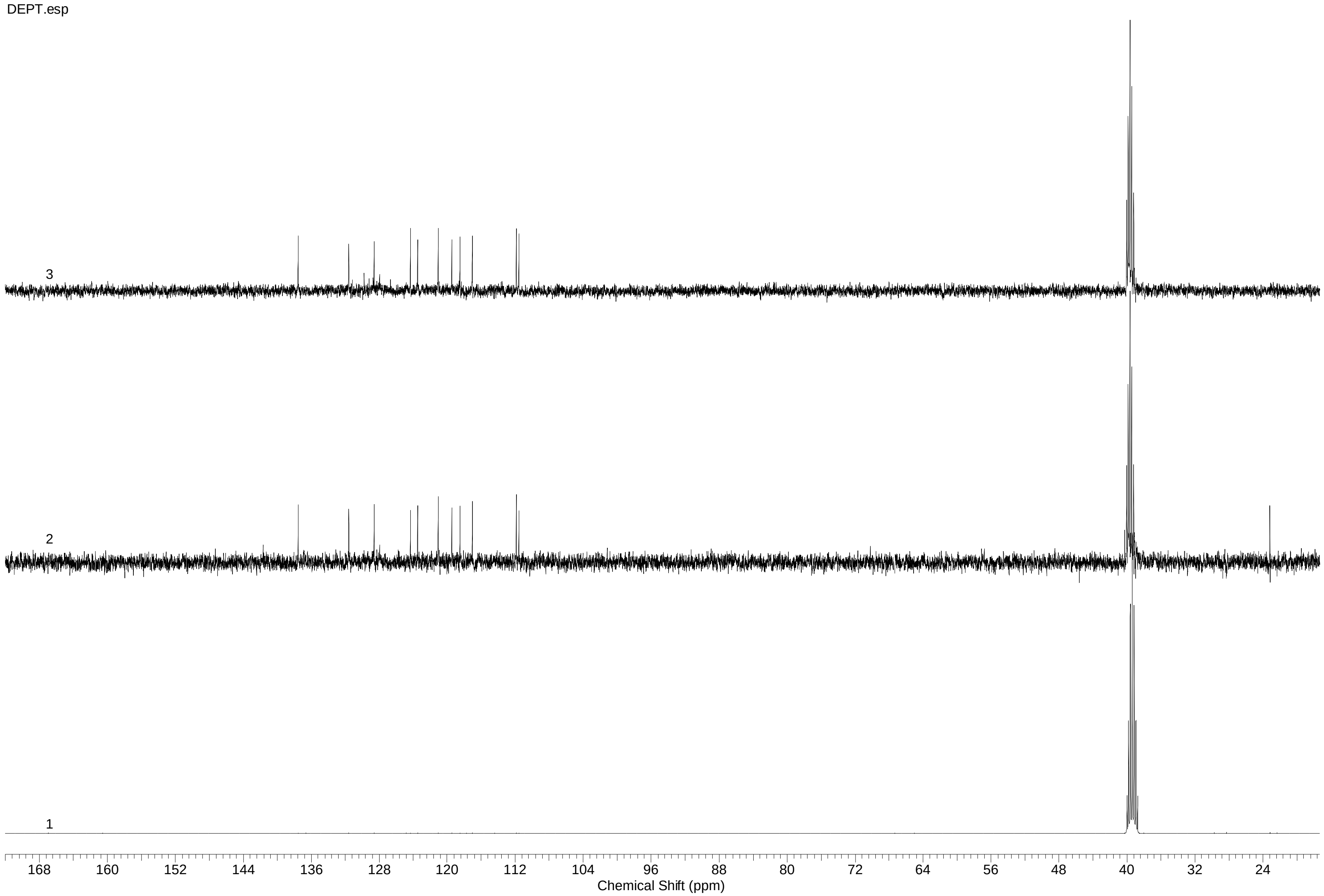

Figure S12. HSQC spectrum (400 MHz) of compound **2** in DMSO-d_6.

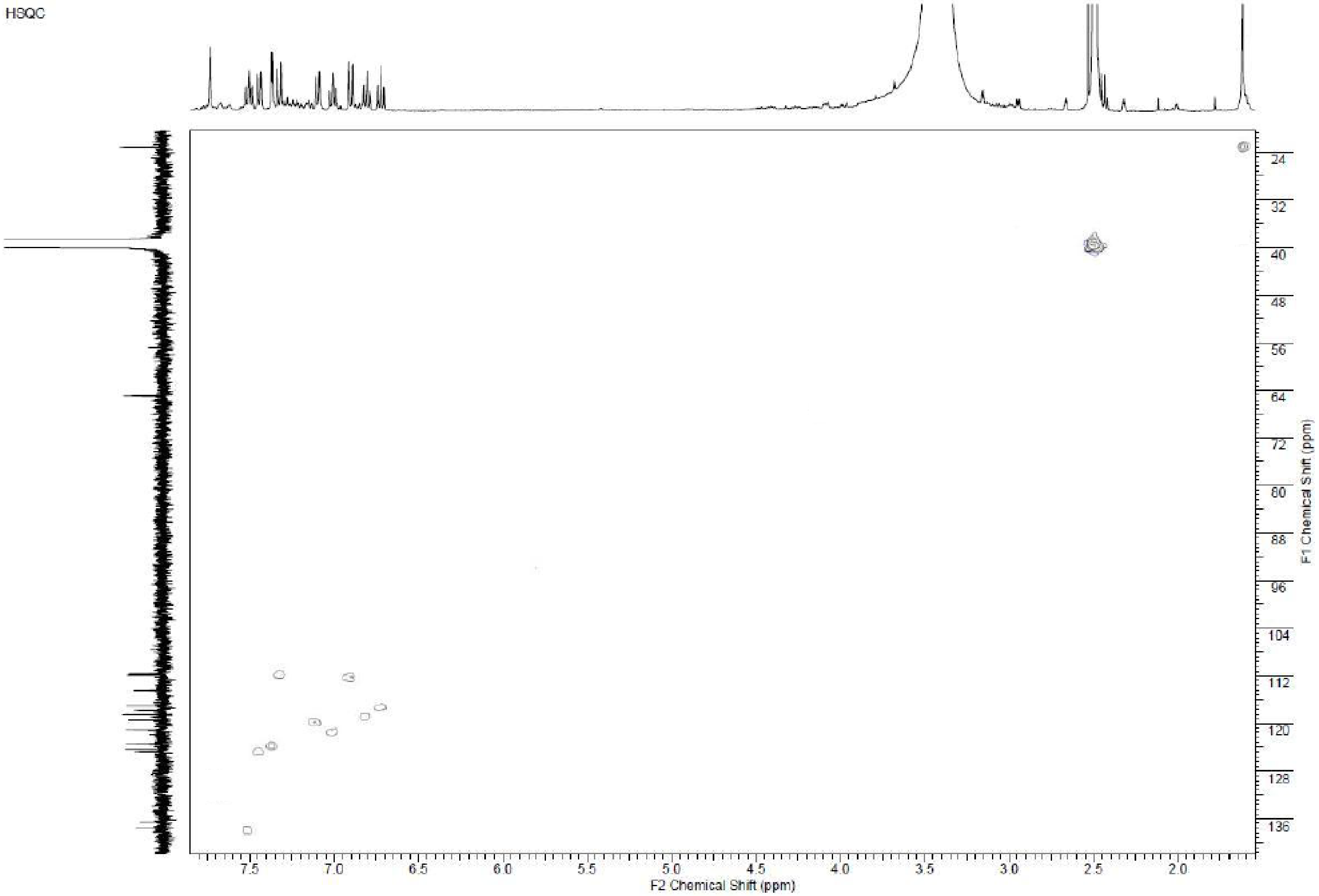

Figure S13. ^{1}H-^{1}H COSY spectrum (400 MHz) of compound **2** in DMSO-d_6.

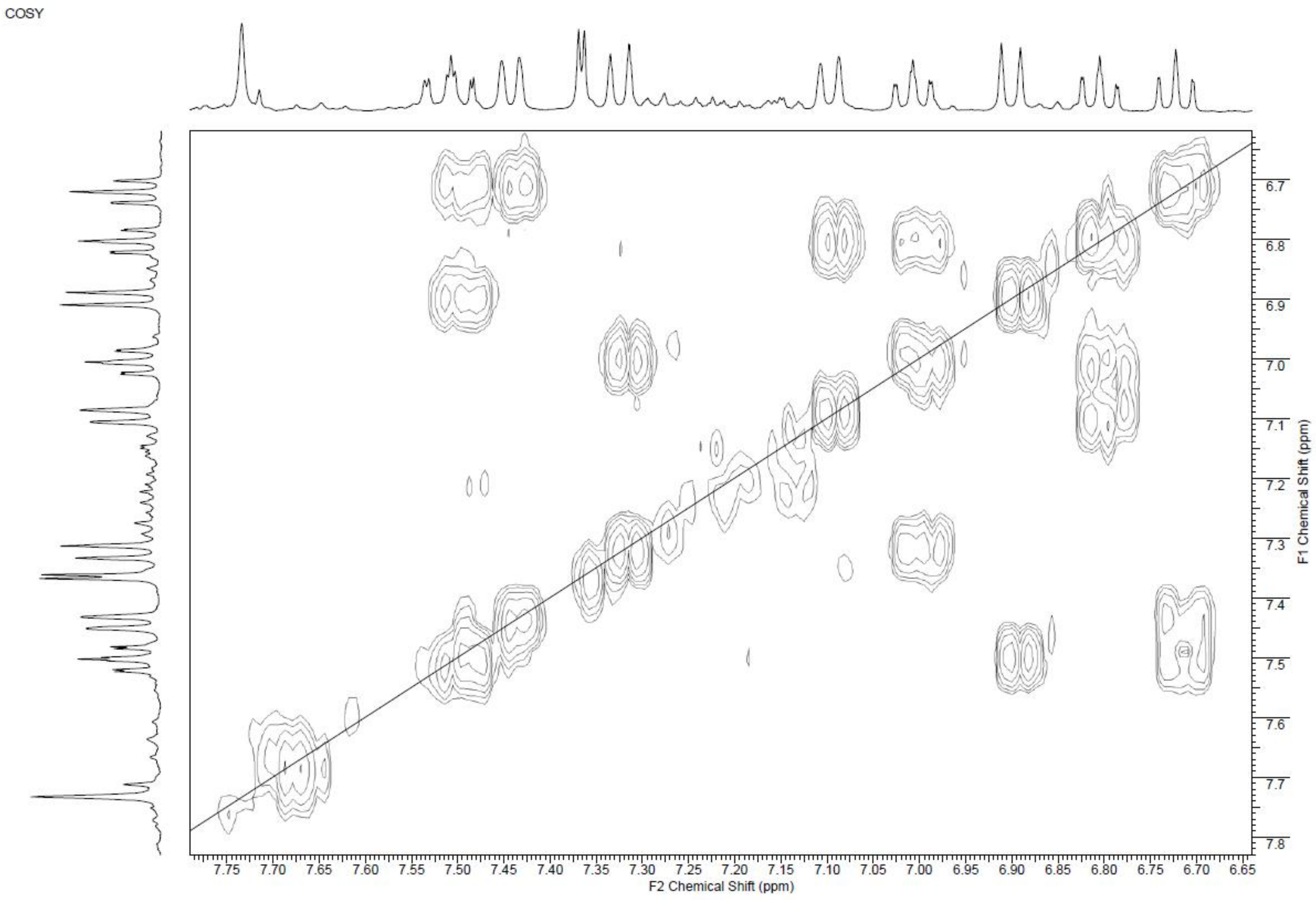

Figure S14. HMBC spectrum (400 MHz) of compound **2** in DMSO-d_6.

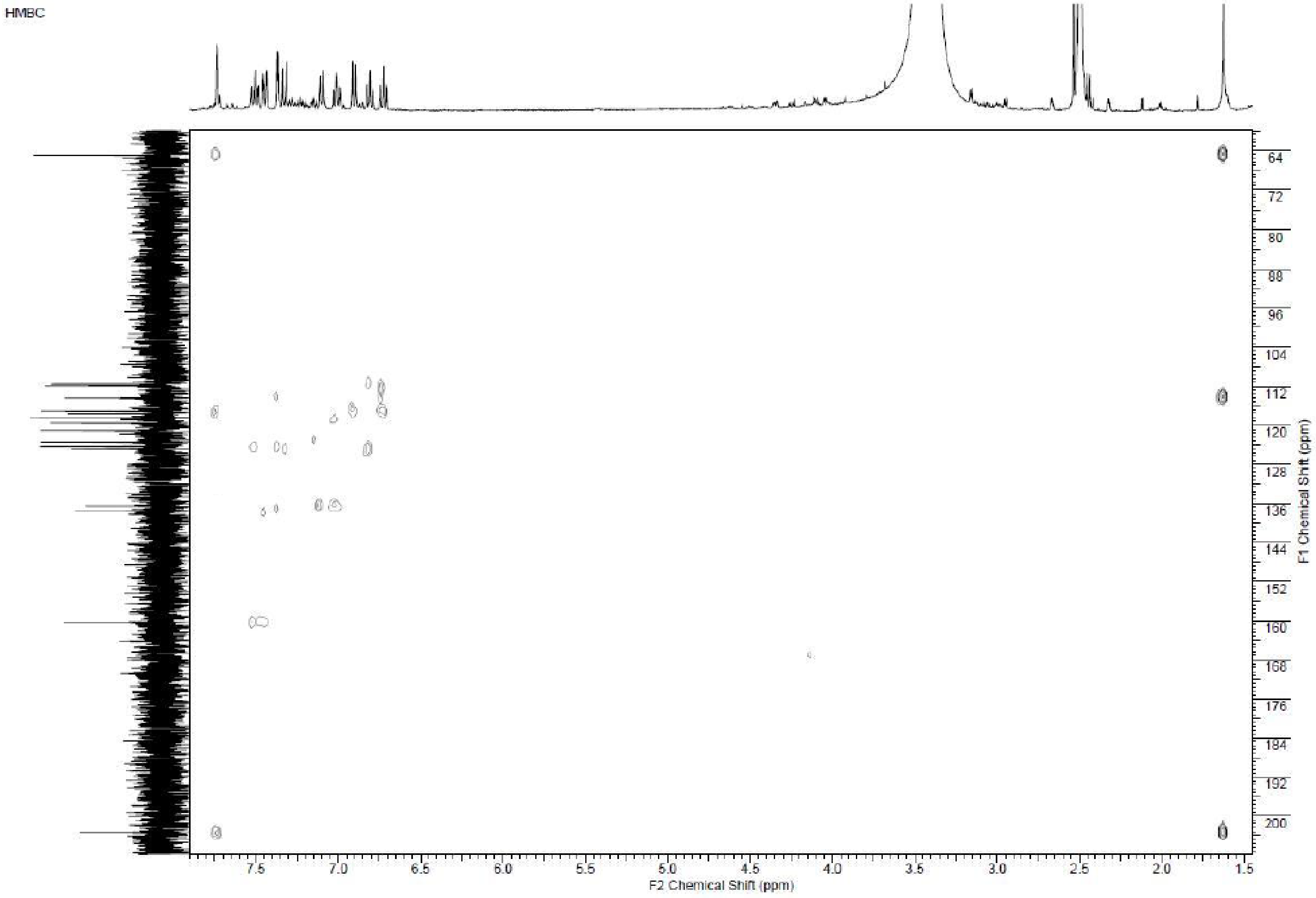

Supplement: Supplementary File 1 — Supplementary Information (PDF, 936 KB) [file marinedrugs-12-02156-s001.pdf]
